# Facebook in general practice: a service evaluation in one health economy

**DOI:** 10.3399/bjgpopen17X101181

**Published:** 2017-10-18

**Authors:** Kevin Moore, Elizabeth Cottrell, Ruth Chambers

**Affiliations:** 1 Medical Student, Keele University School of Medicine, David Weatherall Building, Keele University, Keele, UK; 2 Academic GP, Wolstanton Medical Centre, Keele, UK; 3 NIHR Clinical Lecturer in General Practice, Research Institute for Primary Care & Health Sciences, Keele University, Keele, UK; 4 Clinical Lead, Long Term Conditions Network, West Midlands Academic Health Science Network, Birmingham, UK

**Keywords:** general practice, social media, evaluation

## Abstract

**Background:**

Social media has been utilised in a variety of healthcare settings. While its potential for extending healthcare services is recognised by the NHS, potential pitfalls exist. The place, benefits and practical problems of using Facebook in general practice are unclear.

**Aim:**

To understand the utilisation of Facebook by general practices, whether Facebook provides novel insights when compared to other centrally-hosted feedback platforms, and the prevalence of unofficial Facebook pages.

**Design & setting:**

Eighty-three general practices in North Staffordshire.

**Method:**

Publicly available information and feedback relating to general practices on official and unofficial Facebook sites was examined and compared to other, centrally-hosted feedback platforms (NHS Choices and Patient Satisfaction ratings). Thematic and descriptive analyses were undertaken to understand the nature of the content.

**Results:**

Thirty-one practices had publicly-accessible, practice-owned, official Facebook sites which, overall, had received over 7000 likes. Two had integrated booking systems, 14 allowed reviews and all had accurate practice information. Most remaining practices (41/52) were found to have an unofficial Facebook page.

**Conclusion:**

General practice use of open Facebook pages is variable, but most commonly used to provide generic practice information and for gaining patient feedback. Patient engagement with pages suggests demand for this technology. Risks associated with unmoderated unofficial pages can be mitigated by practices having official pages hosted by the practice with appropriate protocols in place for managing them. Practices need to be supported to better understand meaningful uses of this technology and the potential risks of unofficial practice Facebook pages.

## How this fits in

Social media is widely used both professionally and personally and there is a policy drive to increase the use of technology in primarycare. Use of social media for patients, such as Facebook, by general practice has not been well characterised to date. This service evaluation summarises the use of official social media pages by, and outlines the extent of unofficial pages associated with, general practices, particularly with respect to their common use as a feedback platform. Patient engagement with practice-related social media pages, and the high prevalence of unofficial pages, should prompt general practice teams to consider the place of social media as part of their patient engagement.

## Introduction

Social media has altered the shape of social and commercial communications and become a conduit for rapid networking and information sharing. A recent YouGov survey found that Facebook was the most used social media platform, with 65% of the UK population using it monthly and a 95% usage rate among 16–20 year olds.^1^ Consequently, social media platforms such as Facebook are increasingly recognised as important tools for professional organisations interacting with their target audience.

High profile focus on social media by healthcare students and professionals has primarily been negative; for example, inappropriate use of social media by clinicians and/or medical students.^2,3^ This has led to publication of national guidelines on social media usage.^4–6^ There is much less explicit focus on the potential positive roles of social media in health care despite increasing recognition of the potential for social media in health care, both in UK national policy documents^7,8^ and wider, international literature.^6,9^ However, UK policy documents do not offer support for its implementation. While clinical commissioning group (CCG) support for using social media is provided through the NHS Networks’ *Smart Guides to Engagement* series: *Using Social Media to Engage, Listen and Learn*,^10^ the reach of this information is uncertain.

Potential benefits of social media can include: rapid communication of health messages and service information, and a quick and cheap conduit for feedback.^11^ Equally, though, it can be a platform for rapid and wide dissemination of negative feedback (justified or unjustified), inappropriate content, or misleading or dangerous health information.^11^ Thus, healthcare teams may perceive social media to be an unwelcome threat or burden. Significantly, avoiding engagement with social media does not eliminate the risk of dissemination of negative feedback. NHS Choices, a centrally-hosted UK portal,^12^ has encouraged formal general practice ratings and feedback for some time. However, a concerning development is the ability for members of the community to create unofficial general practice Facebook pages. Practices have no means to moderate content or access to such unofficial pages (unlike their own official pages), leaving the page open for the public (not necessarily registered patients) to relay whatever information they like, possibly without the knowledge of the official organisation itself. Further, the content can be hijacked for unrelated content, such as advertising businesses, falsely giving the impression that the content is endorsed by the practice. While official Facebook pages do not eliminate unofficial sites, by owning an official page (or claiming an unofficial one), practices can increase the chances of a patient reaching official information, as official pages are listed higher than unofficial pages in search results.

While studies have examined the use of social media in primary care, many do not focus solely on the use of Facebook in primary care and often focus is on particular patient and/or carer groups, rather than the general primary care patient population.^11,13,14 ^There are few published papers addressing primary care use of Facebook and none were found that specifically examine the prevalence of unofficial practice Facebook pages. Given that Facebook has an increasing presence in the NHS, this service evaluation aimed to understand the utilisation of Facebook by general practices, to determine whether Facebook provides novel insights when compared to other centrally-hosted feedback platforms, and to understand the prevalence of unofficial Facebook pages in order to inform future use of this technology within the health economy.

## Method

### The service population

This evaluation focused on the 83 general practices within one health economy covering Stoke-on-Trent and North Staffordshire CCGs. Stoke-on-Trent is a mostly urban area with a resident population of 249 000 and a registered population of around 290 000.^15^ It is the 13th most deprived local authority in England.^16^ Newcastle-under-Lyme has a population of 123 900 and Staffordshire Moorlands has a resident population of about 97 100, which covers a combined population of 217 000 registered patients.^15^ The practices included in this study had 463 635 total registered patients with a range of 1242–14 271 registered patients per practice.^17^ Since 2015, a digital expert has been working in the health economy to help practice teams set up and maintain website content and Facebook pages.

### Identification of relevant Facebook pages

The Facebook search function was used to identify official and unofficial Facebook pages for each of the 83 general practices in Stoke-on-Trent and North Staffordshire CCGs. If no results or incomplete results were returned, then a more detailed search using keywords such as "practice name + Stoke-on-Trent + Facebook" in Google was done to try and find relevant practice pages. All searches were undertaken on the same day (1 September 2016) to provide a cross-section of practices’ Facebook presence on that date. While some closed Facebook pages exist, this study only included Facebook pages and information that were publicly available.

### Triangulation of data

NHS patient satisfaction data from 2016 were obtained from the national GP practice survey^18^ and review score and patient comments were obtained from NHS Choices^12 ^in order to establish whether patient feedback obtained through Facebook adds anything to what is already obtainable through centrally-hosted portals, and whether there were any differences between those practices which chose to have open, official Facebook pages and those which did not. The centrally-hosted portals are nationally and freely accessible via the NHS England website.

### Data extraction

On identification of relevant Facebook pages, the official or unofficial status of the pages was noted. Pages were defined as ‘unofficial’ if their header stated that they were unofficial with a contact button to claim the page. 'Official' pages were defined as those which had a ‘verified tick’; verification requires that a strict application process must be followed by those applying. Features of Facebook used (for example, news feeds and online booking) and/or the type of information made available by practices were noted; however, other than identifying the accuracy of the practice information, the content of these was not extracted. Public engagement with identified pages was measured by the total number of ‘likes’, number of Facebook check-ins, number of reviews, and the overall review score. Check-ins were added to Facebook in 2010^19^ and allow the user to broadcast their location and notify other users in their network that they are nearby, usually from options of populated local establishments. It also creates a timeline story on their Facebook feed which allows other users to comment or react. When comments were allowed on an official or unofficial page, all comments entered by users between 1 March 2016 and 1 September 2016 were extracted and practices were ranked by NHS Choices and NHS England patient survey score.

### Data analysis

Extracted free-text comments were categorised as positive, negative, or neutral and by subject matter. Content analysis of the comments was undertaken and emergent themes were defined. No personal details of the people leaving the comments were recorded. All practices in each CCG were ranked according to their NHS patient satisfaction score. Ranks were 1–52 for Stoke-on-Trent and 1–31 for North Staffordshire practices, where 1 was the best satisfaction score and the highest rank for each practice. Ranks were divided into quartiles. Descriptive analyses and comparative analyses were undertaken of review scores from Facebook, NHS Choices, and NHS England in relation to the nature of the comments/reviews left on Facebook.

## Results

### Facebook

**Figure 1.**
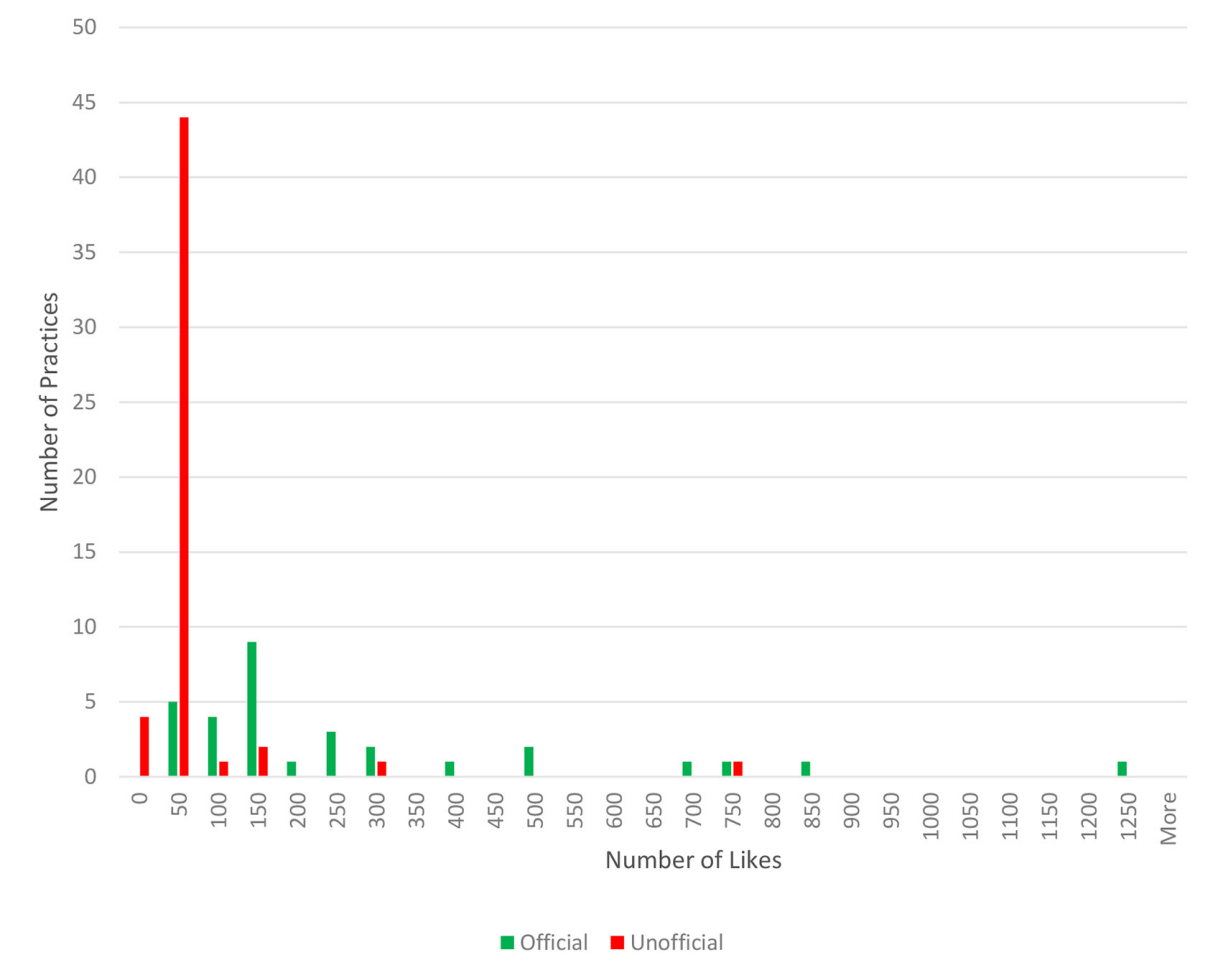
Public engagement indicated by numbers of 'likes' on official and unofficial Facebook pages

Practice engagement with Facebook was variable. Thirteen practices had only an official Facebook page, 18 had both official and unofficial pages, 40 had unofficial pages but no official pages, and 12 had no official or unofficial Facebook presence. Of the 31 practices with official pages, all had accurate practice information, 14 allowed reviews, 11 had a regularly updated Facebook newsfeed, and two had integrated Facebook booking systems. Overall findings for Facebook use and engagement are summarised in Table 1. Eighteen practices had both official and unofficial Facebook pages, one of which had more than one unofficial page. Official pages usually had a greater number of populated fields; for example, unofficial pages did not have a picture of the practice. Two unofficial pages had been vandalised and used for the non-intended purpose of advertising local businesses. One unofficial Facebook page was vandalised in a humorous way that was a pun on the practice’s name. There was a trend towards higher (more positive) review scores on official Facebook pages than on unofficial pages (see Table 1). Generally, the official pages received more likes than unofficial pages (see Figure 1).

**Table 1.** Practice use, feedback and engagement with NHS Choices and Facebook

		NHS Choices	Facebook use	
			Official pages	Unofficial pages
Number of practices		83	31^a^	58^a^
Reviews				
Number of practices allowing reviews		83	14	33
	Number of reviews per practice	5.4	10.7 (SD = 11.5)	6.4 (SD = 4.6)
	Mean review score (SD)	3.6/5 (SD =1.0)	4.25/5 (SD = 0.9)	3.2/5 (SD = 1.7).
User interaction				
Number of likes		N/A	7747 (SD = 279.7)	1358 (SD = 20.7)
Number of check-ins		N/A	4133 (SD = 309.0)	6126 (SD = 141.2)
Mean check-ins per practice (SD)		N/A	159	153

^a^Of which 18 had both unofficial and official pages. SD = standard deviation.

### NHS Choices

NHS Choices scoring was available for all practices and had a mean number of 5.4 reviews per practice and an overall mean review score of 3.6/5 (standard deviation [SD] = 1.0; see Table 1). NHS Choices mean score among practices with official Facebook pages was 3.2/5 (SD = 1.0) and among those without official Facebook pages was 3.9/5 (SD = 1.0). There was no correlation between NHS Choices score and Facebook (unofficial or official) review scores.

### NHS patient satisfaction

Overall NHS satisfaction survey results showed that 87% of practices in Stoke-on-Trent and North Staffordshire had an overall positive rating and 87% of practices with and without an official Facebook page had a overall positive rating. No correlation between NHS patient satisfaction score and Facebook (unofficial or official) review scores was identified.

### Content analysis of free text comments

From all sources, themes emerging from free-text feedback comments were 1) waiting times, 2) GPs and 3) reception staff (see Table 2). Overall 562 comments were positive, 231 were negative and 16 were neutral. Most (87%) positive reviews and comments addressed either GPs or short waiting times. Nearly all (93%) of negative comments were about either reception staff or long waiting times in getting an appointment, or were a combination of both. See Box 1 for examples of comments relating to each theme.

**Box 1.** Examples of comments relating to emergent themes

Theme	Positive	Negative	Neutral
Waiting times	'The online system is great, I regularly need to see the nurse for a contraceptive injection and always can get an appointment within the week, if not the same day, either on the phone or online.' (NHS Choices)	'I took my son who has tonsillitis. 85 minutes to be seen as the GP had gone to lunch and was stuck in traffic!' (NHS Choices)	'All surgeries, it seems, have difficulty getting appointments, but I assume it is because they are so busy.' (NHS Choices)
GPs	'I saw a doctor who was absolutely fantastic and made me feel very comfortable.' (NHS Choices)	'I will always ask for second opinion now if I have to see one of the DRs from here as I [was] wrongly diagnosed early this year.' (Facebook)	'Some of the doctors are better than others.' (Facebook)
Reception staff	'...the staff [reception staff] are also kind, helpful and polite.' (NHS Choices)	'They [reception staff] treat patients with utter disdain and make out that every enquiry is a massive inconvenience.' (NHS Choices)	'The reception staff are okay.' (Facebook)
Others	'The nurses there are brill, worked wonders in my ear.' (Facebook)	'This practice and it seems to rely solely on locums.' (NHS Choices)	'The surgery is always clean and the waiting times are not too bad.' (NHS Choices)

### Comparative analysis

**Figure 2.**
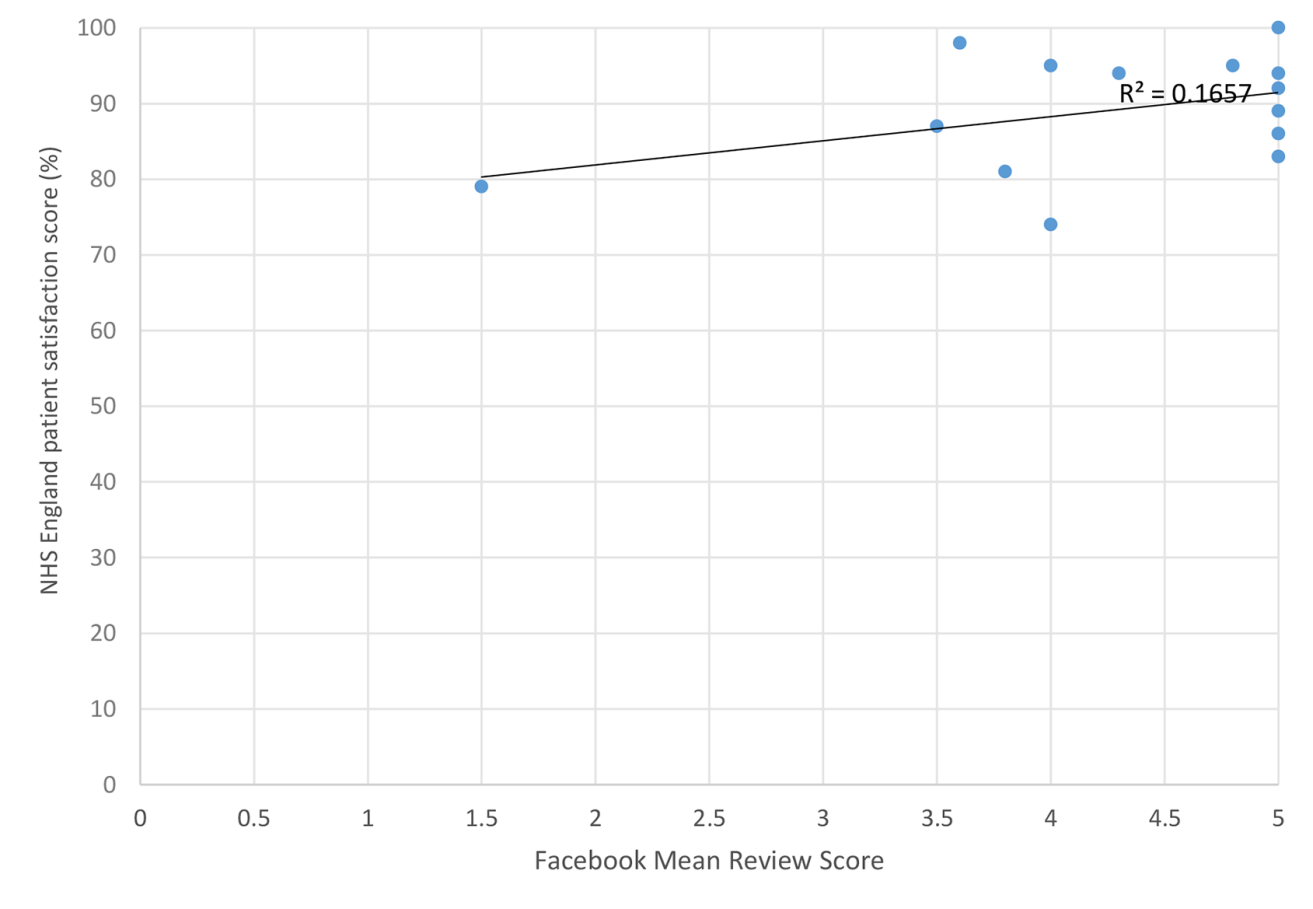
NHS England Patient Satisfaction score (%) versus official Facebook review score.

**Figure 3.**
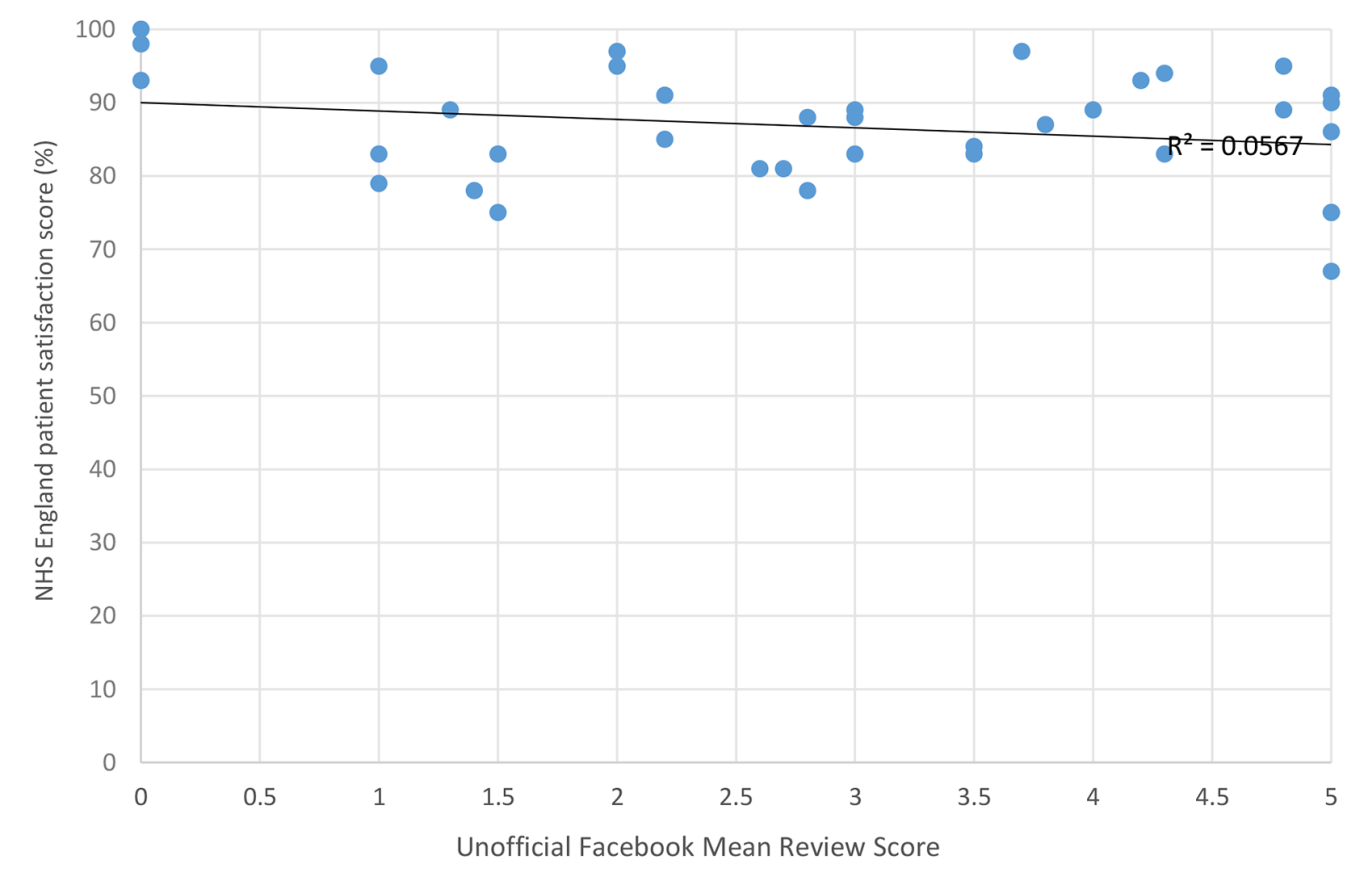
NHS England Patient Satisfaction score (%) versus unofficial Facebook review score.

No relationship was found between positive and negative Facebook and NHS Choices review scores. The existence of official (see Figure 2) and unofficial (see Figure 3) Facebook pages did not seem to clearly relate to NHS England Patient Satisfaction scores. Themes from comments were similar in structure and content on Facebook and NHS Choices (see Table 2 and Box 1).

**Table 2.** Quantitative comparison of feedback found on NHS Choices vs Facebook

Source	NHS Choices	Facebook: official page	Facebook: unofficial page	Total
Theme: waiting times	60	30	33	123
Theme: GPs	301	93	147	541
Theme: reception staff	83	25	30	138
Positive	322	104	136	562
Negative	120	41	70	231
Neutral	6	3	7	16

## Discussion

### Summary

With the internet and social media having almost seamless integration with many people’s everyday lives, there seems to be dissonance between the strong focus on patient-centred care and technology in UK NHS policy documents^4^ and the unstandardised and unclear use of Facebook by general practices. This service evaluation sought to characterise the use of Facebook in general practices which had been offered support to integrate this technology into their service provision, identifying how they used it, what it added in addition to other centrally-hosted feedback platforms, and what the prevalence was of unofficial practice Facebook pages.

Facebook usage by practices reviewed in this study varied widely. While all practices were found to put practice details on to their official pages, some had regularly updated Facebook newsfeeds and a couple had integrated booking systems, the most explicit and measurable function that the practice Facebook pages had were their use as a conduit for patient feedback. No relationship was found between a practice having an official Facebook page and its Facebook review score, NHS Choices score, or NHS patient satisfaction surveys score. In addition, the number of Facebook reviews was higher than the number of NHS Choices reviews. This suggests that Facebook may represent a conduit for feedback not captured elsewhere. Further, patients seemed to engage more with both official and unofficial Facebook sites than with NHS Choices. Reasons may include lack of awareness of NHS Choices and, possibly, the lack of interactivity of this portal. It is not known how widely read the NHS Choices reviews are, or what impact they have on individual practices. The nature of, and much wider usage of, Facebook means that the respective comments, reviews, and check-ins are much more likely to be seen in personal networks with targeted information.

The high levels of engagement with some Facebook pages, suggests that there is an appetite among the public for healthcare organisations to have a social media presence and that information disseminated through this route has potential to reach a large number of people. However, it seems that, in general, the potential benefits for Facebook have not been maximised. Considering that previous work has highlighted a lack of clarity about the role of, confidence in, and use of social media in a primary care setting, as well as discordance between patients' and healthcare professionals' perceptions of the latter's healthcare-related use of social media,^20,21^ further efforts may be required to better support the use of this approach and improve communication between service providers and their patients.

Finally, the high prevalence of unofficial pages identified was a concern, from the point of view of practice professional integrity and the possible impact such sites may have on patients. Not all of these unofficial pages will have been set up with malicious intent; indeed, this seemed to be rare in the sample examined in this evaluation. Rather, they may be inadvertently created by Facebook users checking-in to a practice that does not already have a Facebook presence. This may result in duplications of pages. It is possible, although uncommon, for Facebook users to actively set up unofficial pages to use however they wish.

## Strengths and limitations

This service evaluation addressed a single health economy with a large population of around half a million patients. Therefore, it has provided a good snapshot of how Facebook is being used in current general practice. However, limitations include the use of only one author to undertake the data extraction and analysis, as only one opinion was used to qualitatively assess the comments as positive/negative/neutral, and the inability to access private or closed Facebook groups (hence the number and use of official Facebook sites are likely to be underestimated). Limiting the analysis to only publically available data also prevented measurement of the use of the non-feedback functions of Facebook. The presence of a digital expert who was employed in the local health economy to support the use of Facebook will limit the applicability of the results to other areas which lack such support. The heavily deprived nature of Northern Staffordshire also results in the information obtained in this evaluation being less relevant to areas with a different socioeconomic status. Further, it is not clear how relevant the findings are to more commercial providers of health care, for example, the private sector in the UK or other healthcare systems abroad. Finally, given the large variation in size of practices included, results may have been skewed by content from the larger practices. However, the larger practices did not necessarily produce the higher number of likes; for example, one practice with 3344 registered patients had received 1228 likes.

## Implications for practice

In Northern Staffordshire, it appears that existing Facebook users have the desire and willingness to communicate with and about general practices using this type of social media. The steps required to either create or claim an official page with underpinning administration and ongoing maintenance are minimal and practices who have engaged with the technology have reported that this is straightforward with adequate protocols in place.^21^ In addition to considering the administrative burdens and risks to reputation associated with Facebook use, general practices should consider the real risk of unofficial Facebook pages being set up and commanding public engagement without their knowledge. Evidence was found of unofficial pages having been vandalised and used for the non-intended purpose of advertising local businesses; further, there was a trend towards less positive feedback on unofficial Facebook pages. Thus, it is suggested that practices consider the value of hosting at least an official landing page which, as a minimum, would act as a directory listing for a practice. This would serve a subsidiary function of preventing unmoderated, inaccurate, and inappropriate information within unofficial pages being prominent in search results; although, within the examples found, content was generally not harmful, these have potential to seriously damage the reputation of a practice if interpreted as being practice-endorsed. As technology increases in prominence throughout healthcare providers' service offerings, there are many opportunities that can be utilised by practices, from spreading health information through to online appointment booking (used by some practices included in this study). Engaged practices would need to be prompt at dealing with any negative issues such as negative or inflammatory comments posted on their Facebook site; however, existing practice users have developed protocols aligned with their complaints procedures to address these. Other issues such as cost and implementation processes are real, but not insurmountable.

In conclusion, use of Facebook is variable but public engagement with pages suggests demand for this technology. Risks associated with unofficial pages arise from their unmoderated status and can be mitigated by practices having official pages hosted by the practice with appropriate protocols in place for managing them. Practices need to be supported to better understand meaningful uses of this technology and the risks of unofficial practice Facebook pages.
